# Diagnosis and treatment of right ventricular dysfunction in patients with COVID-19 on veno-venous extra-corporeal membrane oxygenation

**DOI:** 10.1186/s13019-022-02028-w

**Published:** 2022-11-06

**Authors:** Maziar Khorsandi, Jeffrey Keenan, Mackenzie Adcox, Ariyan Tabesh, Jenelle Badulak, Jay Pal, Michael Mulligan

**Affiliations:** 1 Division of Cardiothoracic Surgery, University of Washington Medical Center, 1959 NE Pacific St, Seattle, WA 98195 USA; 2 Division of Pulmonary, Critical Care and Sleep Medicine, University of Washington Medical Center, Seattle, WA USA

**Keywords:** COVID-19, Extra-corporeal membrane oxygenation, Right ventricular failure, Right ventricular assist

## Abstract

**Background:**

Veno-venous (VV) extracorporeal membrane oxygenation (ECMO) is an effective, but highly resource intensive salvage treatment option in COVID patients with acute respiratory distress syndrome (ARDS). Right ventricular (RV) dysfunction is a known sequelae of COVID-19 induced ARDS, yet there is a paucity of data on the incidence and determinants of RV dysfunction on VV ECMO. We retrospectively examined the determining factors leading to RV failure and means of early identification of this phenomenon in patients on VV ECMO.

**Methods:**

The data was extracted from March 2020 to March 2021 from the regional University of Washington Extracorporeal Life Support database. The inclusion criteria included patients > 18 years of age with diagnosis of COVID-19. All had already been intubated and mechanically ventilated prior to VV ECMO deployment. Univariate analysis was performed to identify risk factors and surrogate markers for RV dysfunction. In addition, we compared outcomes between those with and without RV dysfunction.

**Results:**

Of the 33 patients that met inclusion criteria, 14 (42%) had echocardiographic evidence of RV dysfunction, 3 of whom were placed on right ventricular assist device support. Chronic lung disease was an independent risk factor for RV dysfunction (*p* = 0.0002). RV dysfunction was associated with a six-fold increase in troponin I (0.07 ng/ml vs. 0.44 ng/ml, *p* = 0.039) and four-fold increase in brain natriuretic peptide (BNP) (158 pg/ml vs. 662 pg/ml, *p* = 0.037). Deep vein thrombosis (DVT, 21% vs. 43%, *p* = 0.005) and pulmonary embolism (PE, 11% vs. 21%, *p* = 0.045) were found to be nearly twice as common in the RV dysfunction group. Total survival rate to hospital discharge was 39%. Data trended towards shorter duration of hospital stay (47 vs. 65.6 days, *p* = 0.15), shorter duration of ECMO support (21 days vs. 36 days, *p* = 0.06) and improved survival rate to hospital discharge (42.1% vs. 35.7%, *p* = 0.47) for those with intact RV function compared to the RV dysfunction group.

**Conclusions:**

RV dysfunction in critically ill patients with COVID-19 pneumonia in common. Trends of troponin I and BNP may be important surrogates for monitoring RV function in patients on VV ECMO. We recommend echocardiographic assessment of the RV on such patients.

## Background

The outbreak of COVID-19 shocked many health care systems across the world, taking millions of lives [1–5]. During H1N1 and MERS-CoV outbreaks, Mechanical circulatory support (MCS) had been utilized in severe cases refractory to optimal ventilator therapy and prone positioning with some success [6]. Although MCS has proved to be an effective salvage strategy in the immediate term in severe COVID-19 induced ARDS, the mortality rate has remained very high [6]. COVID-19 proved to have caused not only ARDS and respiratory failure but also right ventricular dilatation and dysfunction [7]. Right ventricular (RV) dilatation has been associated with significantly worse outcome in COVID-19 patients [7]. This phenomenon, likely reflects the increased afterload on the RV from secondary pulmonary hypertension as well as primary cardiomyopathic insult from the viral infection, often rendering VV ECMO strategy per se ineffective due to the resultant low cardiac output and end organ dysfunction. Utilization of right ventricular assist devices (RVAD) with oxygenators can mitigate both respiratory and right heart failure with some reported success [8]. Earlier case series have reported RV dilation to be present in as much as 41% of patients with COVID-19 induced ARDS and 27% of patients had impaired RV function [9] and identified C-reactive protein (CRP) and D-dimer as associated biomarkers of RV dysfunction [9–11].

**Fig. 1:**
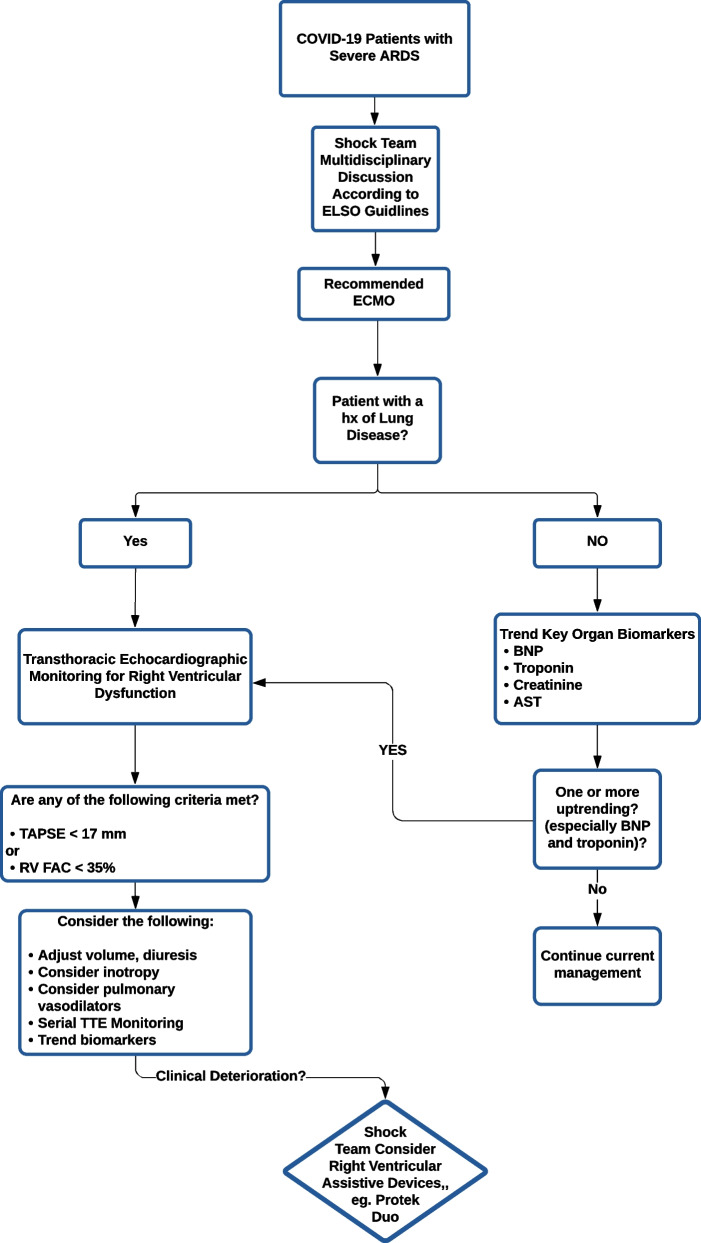
Flow chart for early diagnosis and management of right ventricular dysfunction in COVID-19 patients who require VV ECMO

In this retrospective study we assessed our outcomes of MCS for COVID-19 patients in a regional high-volume center with a large geographic catchment area encompassing the pacific northwest of the United States. The aim of this study is to assess the effectiveness of VV ECMO in supporting patients with severe ARDS associated with COVID-19 as well as incidence of RV failure. We paid particular attention to the trends of the surrogate laboratory markers predicting RV dysfunction requiring inotropic and RVAD support. We trended end organ dysfunction, ICU length of stay, duration of mechanical circulatory support (if applicable), any complications related to right heart failure, and survival rate to hospital discharge. We also developed an algorithm (Fig. 1) to simplify early diagnosis of RV dysfunction and the need escalation of support (including placement of RVAD) before end organ dysfunction sets in.

## Materials and methods

The data was extracted from March 2020 to March 2021 from the regional University of Washington Extracorporeal Life Support database. All subjects were adults, 18 years or older, with COVID-19 infection, confirmed via reverse transcriptase-polymerase chain reaction (RT-PCR) for SARS-CoV-2 virus (i.e. COVID-19) with acute respiratory distress syndrome (ARDS). They all had refractory hypoxemia, requiring Veno-venous Extracorporeal Membrane Oxygenation (VV ECMO) hospitalized at the University of Washington Medical Center or Harborview Medical center in Seattle, Washington were included in this study. ARDS was diagnosed using the Berlin criteria [12] as follows: 1. Presence of acute hypoxemic respiratory failure, 2. Worsening respiratory symptoms within 7 days, 3. Cardiac failure not the etiology for respiratory failure and 4. Presence of bilateral opacities. The observation of bilateral pulmonary infiltrates in this study were done on computed tomography (CT) scan in keeping with the Berlin criteria for ARDS. Our patient cohort included patients across the WWAMIO region (Washington, Wyoming, Alaska, Montana, Idaho, and Oregon), covering a population of approximately 11.8 million people according to the U.S Census Bureau and approximately 1/5th of the USA landmass.

Institution Review Board (IRB) was obtained from the University of Washington research review board. Patients’ charts were accessed and reviewed. Information regarding basic demographic background (age, race, gender) as well as body mass index (BMI), comorbidities, and smoking history were gathered. The serial pre-VV ECMO cannulation arterial blood gases (ABG) and ventilator settings were obtained to assess whether the severity of ARDS and higher ventilator settings were correlated with RV dysfunction. Key organ functions were evaluated using surrogate peak biomarkers values for creatinine, aspartate aminotransferase (AST), troponin I, and brain natriuretic peptide (BNP). Transthoracic echocardiography (TTE) data for all patients that had a TTE performed during their hospital course were reviewed. RV dysfunction was defined as tricuspid annular plane systolic excursion (TAPSE) of < 17 mm or right ventricular fractional area change (RV FAC) of < 35% in accordance with the criteria developed by the American Society of Echocardiography [13]. Accordingly, patients were categorized into two groups: no RV dysfunction and RV dysfunction. The patients with no TTE studies were presumed to have no RV dysfunction and were placed in the no RV dysfunction group accordingly. The criteria for percutaneous right ventricular assist device (RVAD) support were RV dysfunction leading to hemodynamic instability and/or progressive end organ (liver and renal) dysfunction, and available ECMO capacity in the university hospital capable of managing patients with MCS. All RVADs were placed percutaneously in the hybrid suite and using the right internal jugular vein or left subclavian vein approach (Tandem Heart Protek Duo, dual lumen cannula). Additionally, the severity of lung injury was assessed using pre-VV ECMO ventilation settings, PaO2/FiO2 ratio, and ABGs. Hospital course and outcomes were evaluated by comparing rates of complications, survival to discharge, and duration of mechanical and circulatory support.

### Statistical analysis

Univariate analysis was performed to identify the prognostic indicators. All data were compared across the two groups by calculating statistical p-values using the chi-squared test for qualitative parameters and the t-test for the quantitative measure. The value of 0.05 or less was deemed statistically significant.

## Results

We identified 33 patients that met the aforementioned inclusion criteria, Baseline demographic characteristics, as well as risk factors and comorbidities, are summarized in Table 1. The mean age was 49 ± 9 years. 73% (n = 24) of patients were male. The majority of our patients were non-Caucasian (79%, n = 26). This was a significant finding as the WWAMIO region is overwhelmingly populated by white Caucasians, indicating ethnic minorities are more severely affected by COVID-19.

**Table 1:** Patient related factors and comorbidities and the prevalence of right ventricular dysfunction

Demographics and PMH	Total, n = 33	No RV dysfunction, n = 19	RV dysfunction, n = 14	*P* value
Mean age (years) ± STDV	48.7 ± 9.2	50.4 ± 7.7	46.5 ± 10.5	
Mean BMI ± STDV	30.2 ± 3.5	30.9 ± 3.4	29.3 ± 3.6	
Male	72.7%	73.7%	71.4%	
Non-White	78.8%	73.7%	85.7%	
DM %	36.36%	42.11%	28.60%	0.109
HTN %	33.33%	42.11%	21.43%	0.0104
Smoker %	35.48%	29.41%	42.86%	0.109
Lung disease (OSA, Asthma, COPD) %	27.27%	15.79%	42.86%	0.0002

### Risk factors for right ventricular dysfunction

RV dysfunction was identified in 42% (n = 14). 24% (n = 8) never had echocardiographic studies. History of lung disease including obstructive sleep apnea, asthma, and COPD were 2.7 times more prevalent in the RV dysfunction group than the no RV dysfunction group (*p* = 0.00021). Amongst the RV dysfunction cohort, 3 received percutaneous RVAD support in the form of percutaneous dual lumen cannula through the right internal jugular vein inserted under fluoroscopic guidance. Please see echocardiographic assessment on Table 2.

**Table 2:** Echocardiographic assessment

Echocardiographic parameters	Total, n = 33	No RV dysfunction, n = 19	RV dysfunction, n = 14
Mean TAPSE	1.749	1.975	1.619
Mean RV FAC %	25.19	43	23.82
Mean PAP (mmHg)	51.21	40.133	55.957
Mean LVEF %	61.89	64.87	59.55

Troponin and BNP were shown to be 6 times and 4 times higher in the RV dysfunction cohort, whereas AST and creatinine levels were not observed to be statistically different between the two groups (see Table 3).

**Table 3:** Surrogates for end organ dysfunction

Surrogates for end organ dysfunction	Total, n = 33	No RV dysfunction, n = 19	RV dysfunction, n = 14	*P* value
Mean pH	7.310	7.301	7.321	0.555
Mean pCO_2_	66.313	66.833	65.643	0.867
Mean pO_2_	79.250	81.222	76.714	0.632
Mean HCO_3_	33.097	32.412	33.929	0.651
Mean PEEP	13.750	14.278	13.071	0.287
Mean FiO_2_	88.44%	91.11%	85.00%	0.191
Mean tidal volume cc/kg	5.210	5.359	5.039	0.352
PaO_2_/FiO_2_ ratio	90.51	89.1	92.34	0.752
Mean peak AST	539.2	583.2	479.6	0.841
Mean peak creatinine	2.3	2.2	2.5	0.710
**Mean peak BNP**	**433.3**	**158.5**	**662.3**	**0.037**
**Mean peak troponin I**	**0.24**	**0.07**	**0.44**	**0.039**

Bold indicate statistical significance

### Complications

We observed that 69% (n = 23) of our patients developed ventilator-associated pneumonia. Hemorrhagic complications took place in 51% (n = 17), followed by renal failure requiring dialysis in 36% (n = 12), thromboembolic phenomenon in 30% (n = 10) urinary tract infections in 21% (n = 7), bacteremia in 21% (n = 7), pneumothorax in 15% (n = 5) and heparin-induced thrombocytopenia (HIT) in 15% (n = 5).

A more in-depth analysis revealed that RV dysfunction was associated with a two fold increased risk of pulmonary embolism (21.42% vs. 10.53%, *p* = 0.04) and two fold higher risk of thrombosis/deep venous thromboses (42.85% vs. 21.05%, *p* = 0.0046). Table 4 summarizes complication rates as related to RV dysfunction.

**Table 4:** Complication rates as related to presence or absence of RV dysfunction

Complications	Total, n = 33	No RV dysfunction, n = 19	RV Dysfunction, n = 14	P Value (chi-square test)
Hemorrhage	51.50%	52.26%	50%	0.815
Heparin induced Thrombocytopenia	15.15%	15.79%	14.29%	0.783
Pneumonia	69.69%	68.42%	78.57%	0.283
**Thrombosis/DVTs**	**30.30%**	**21.05%**	**42.85%**	**0.005**
Urinary tract infection	21.21%	21.05%	21.43%	0.950
Bacteremia	21.21%	21.05%	21.43%	0.953
Pneumothorax	15.15%	15.79%	14.29%	0.783
**Pulmonary embolism**	**15.15%**	**10.53%**	**21.42%**	**0.045**

Bold indicate statistical significance

Total of 9% (n = 3) patients required placement of percutaneous RVAD. All were female, aged between 31 and 46 years old. They all had a history of asthma, and one also had a distant history of H1N1 infection for which she had undergone intubation and ventilation. All three patients exhibited severe RV dysfunction characterized by RV FAC < 35% and elevated pulmonary artery pressures (PAPs). Two patients were able to recover from ARDS and were discharged to the inpatient rehabilitation service and later discharged to home. The other did not show any evidence of cardiopulmonary recovery despite a prolong period of support and died when support was withdrawn.

The overall survival rate in our cohort study of COVID-19 VV ECMO was 39% (n = 13). The RV dysfunction group had a longer duration of mechanical ventilation, mechanical circulatory support, and longer total hospital stay. However, these findings did not reach statistical significance (see Table 5).

**Table 5:** Outcome data as related to presence or absence of significant RV dysfunction

Outcomes	Total, n = 33	No RV dysfunction, n = 19	RV dysfunction, n = 14	*P* value
Survival to discharge %	39.4%	42.1%	35.7%	0.466
Mean duration of mechanical ventilation (days)	36.3	32.4	41.7	0.182
Mean duration of mechanical circulatory support (days)	27.3	20.9	35.9	0.062
Mean duration of total hospital stay (days)	54.9	47.0	65.6	0.153

## Discussion

RV injury, dilation and dysfunction as a result of COVID-19 pneumonia is a well-recognized complication of this condition [7] and previous studies have objectively examined this phenomenon through echocardiography [11, 14] and magnetic resonance imaging (MRI)(15). It has been demonstrated that presence of RV dysfunction is a significant adverse prognostic indicator in severely ill patients with COVID-19 [10, 15–17]. We identified that an overwhelming majority (78.8%) of the patients admitted needing VV ECMO for COVID-19 were of non-white ethnic background. This is significant in the WWAMIO region with predominantly white population. However, higher incidence and higher morbidity and mortality rates in the non-Caucasian ethnic minorities from COVID-19 is a well reported phenomenon that could possibly be explained by socioeconomic disparities and lack of access to health care [18, 19].

In this study of COVID-19 patients on VV ECMO, 42% of cohort demonstrated echocardiographic evidence of clinically significant RV dysfunction. This is remarkable as it confirms the previously shown high incidence of RV dysfunction within patients with ARDS secondary to COVID-19 reported by other studies [20–22]. Additionally, we identified that pre-existing chronic lung disease increases the risk of RV dysfunction by nearly threefold. As such these patients should be more attentively monitored for evidence of RV dysfunction using TTE, particularly with TAPSE and RV FAC measurements. A previous study by Lan et al. [10], demonstrated elevation in serum markers of D-Dimer and C-reactive protein (CPR) as surrogates of RV dysfunction. We do acknowledge that, while it is not possible to conclusively say which entity (RV failure or Troponin I/BNP elevation) preceded which, it is important to emphasize that Troponin I and BNP are highlighted as potential screening tools to detect and preempt RV failure earlier in its course, and any rise in these lab values should prompt trending these values in time and performing further investigation looking for evidence of RV dysfunction before “full on” RV failure and end organ dysfunction has set in. In this study, we identified that serum Troponin I and BNP were strong indicators of RV dysfunction. Our data showed troponin I elevation was sixfold and BNP elevation was fourfold higher in the RV dysfunction cohort as compared to the non-RV dysfunction cohort. Indicating that up-trending levels of these two markers should prompt physicians to monitor patients for RV dysfunction using TTE. We also recommend that any patient who requires VV ECMO due to COVID-19 should get a baseline and a follow-up TTE to monitor RV function.

Notably also we identified significantly higher thromboembolic phenomenon in patients after they developed RV dysfunction. It is known that COVID infection is a thrombogenic state. However, we also pustulate that higher venous pressures leading to greater stagnation of blood, predisposing patients to thromboembolic risk.

Furthermore, we report survival to hospital discharge of 39% amongst our COVID-19 patients who required VV ECMO. Our study could not further confirm findings by previous studies that reported higher mortality rates in patients with RV dysfunction. However, our study did demonstrate a trend towards increased length of hospitalization, as well as duration on mechanical and circulatory support in the RV dysfunction cohort. It is unknown whether RVAD support of patients with RV failure due to COVID ARDS improves mortality, and furthermore unknown if RVAD rescue of patients on VV ECMO with a failing RV is a viable option. This question is ripe for further study as some preliminary data suggest impressively high rates of survival in patients supported using RVADs. Mustafa et al. [23], reported a study of 40 consecutive patients undergoing VV ECMO for COVID-19 related respiratory failure. They reported that 29 (73%) were weaned off from ECMO and were eventually discharged from the hospital without need for further oxygen therapy. However, this study did not report any significant RV failure or the need for RVAD in of their patients, which is a unique feature of our report.

Based on the findings presented in this study we devised a flowchart algorithm (see Fig. 1) for evaluation, monitoring and management of COVID-19 patients on VV ECMO with respect to RV dysfunction.

## Conclusions

In conclusion, we identified that a significant proportion of patients with COVID-19 infection requiring VV ECMO develop RV dysfunction. There is significant predilection of this phenomenon for ethnic minorities. Troponin I and BNP were identified as early biomarkers of RV dysfunction. This study reinforces the standard of care of obtaining baseline and serial TTE in these critically ill COVID-19 patients on VV ECMO. A close and systematic assessment during the ECMO Support with biomarkers and echocardiography might predict impairment and evolution towards RV failure and properly change the therapeutic approach including percutaneous RVAD support.

## Limitations

In our study not all patients had echocardiography, in whom laboratory surrogates were utilized as markers of RV failure. This study is inherently limited by limited number of subjects as well as its retrospective nature.

## Data Availability

Data could be requested and accessed through the corresponding author mkhors@uw.edu (M. Khorsandi).

## Abbreviations

ECMO: Extra-corporeal membrane oxygenation
RVAD: Right ventricular assist device
RV: Right ventricle
CT: Computed tomography
MCS: Mechanical circulatory support
ABG: Arterial blood gas
ARDS: Acute respiratory distress syndrome
CRP: C-reactive protein
BNP: Brain natriuretic peptide
TAPSE: Tricuspid annular plane systolic excursion
FAC: Fractional area change
PAP: Pulmonary artery pressures
TTE: Transthoracic echocardiogram
WWAMIO: Washington, Wyoming, Alaska, Montana, Idaho, and Oregon
